# Prevalence of Congenital Heart Defects in Individuals With Down Syndrome in Saudi Arabia: A Systematic Review and Meta-Analysis

**DOI:** 10.7759/cureus.31638

**Published:** 2022-11-18

**Authors:** Roweim Sharaf, Wallaa Garout, Renad Sharaf

**Affiliations:** 1 General Practice, King Abdulaziz University Hospital, Jeddah, SAU; 2 Paediatrics, King Abdulaziz University Hospital, Jeddah, SAU; 3 Medicine and Surgery, King Abdulaziz University Faculty of Medicine, Jeddah, SAU

**Keywords:** paediatrics, meta-analysis, saudi arabia, down syndrome, congenital heart defects

## Abstract

Patients with Down syndrome (DS) are commonly diagnosed with congenital heart disease (CHD), which is the leading cause of mortality in this group during the first two years of life. This systematic review and meta-analysis aims to review the current publications to assess the pooled prevalence of overall CHDs in individuals with DS in KSA. We conducted the search on the databases PubMed, EBSCO, Scopus, Web of Science through Clarivate, and Google Scholar using Boolean operators and various keywords. The Rayyan - Intelligent Systematic Reviews website (https://www.rayyan.ai/) was used for citation management and MedCalc® Statistical Software version 20.115 was used for the quantitative data synthesis (MedCalc Software Ltd., 2022, Ostend Belgium). We initially retrieved a total of 402 citations from the primary search and 10 articles were finally included after title screening and full-text assessment. A total of 1590 subjects were enrolled in the pooled analyses. The pooled prevalence of CHDs was found to be 66.1% (95% CI: 57.2% to 74.5%). There was significant heterogeneity (I2 = 92.2%), and inspection of the funnel plot shows the symmetrical distribution of plotted data. According to our study, 66% of DS patients in Saudi Arabia had one or more congenital cardiac defects. Due to the significant inter-study heterogeneity, the reliability of our results is, nevertheless, limited. We advise conducting more research to provide better data for determining the prevalence of CHD.

## Introduction and background

Down syndrome (DS), a genetic condition with an estimated worldwide frequency of 1/600-1/1000 live births [1], is marked by mental disability, developmental delay, a distinctive facial appearance, congenital abnormalities, and hypotonia at infancy [2]. However, it is predicted that 1 in 554 live births in Saudi Arabia results in DS [3]. The likelihood of having a child with DS rises with maternal age; as a result, at the ages of 40 and 50, the chances are 1 in 97 and 1 in 6, respectively [4].

Over the last several decades, there has been an increase in the incidence of DS [5]. Along with the rise in frequency, instances of congenital heart anomalies in developed countries have shown a marked improvement in life expectancy [6]. In fact, even though postoperative morbidity is still widespread, DS does not significantly increase the risk of death after the majority of cardiac surgeries [7].

On the contrary, infants with DS who have heart defects are more likely to die in their first year of life in developing nations [8]. Although these newborns need comparable therapy as non-DS babies, access to care is a significant barrier. Therefore, congenital heart disease (CHD) is a prominent factor in patients in this category who die young [9]. In the DS population, CHD is the primary cause of death and morbidity in the first two years of life [10], and 40% to 63.5% of DS patients have CHD [2,11,12].

The profile and nature of these CHDs may vary depending on the various geographical regions of the globe, according to some research. Indirect evidence of the causative impact of environmental variables is provided by recent studies conducted in Norway, which also demonstrate seasonal change in the incidence of DS and birth abnormalities [13]. Genetic factors do not show seasonal variation [14]. To the authors’ knowledge, no previous systematic reviews reported the prevalence of CHD among individuals with DS in the Kingdom of Saudi Arabia (KSA).

## Review

Methodology

Study Design

This systematic review and meta-analysis was conducted following the Preferred Reporting Items for Systematic Reviews and Meta-Analyses (PRISMA) 2020 recommendations [15].

Study Duration

This study was conducted in the period from September 1, 2022, to September 25, 2022.

Search Strategy

We conducted the search using the databases PubMed, EBSCO, Scopus, Web of Science through Clarivate, and Google Scholar. The search was performed using keywords, MeSH terms, and the Boolean operators AND and OR. The search keywords included down syndrome; trisomy 21; Mongolism; 47,XY,+21; Trisomy G; 47,XX,+21; Down's Syndrome; Downs Syndrome; Syndrome; Down's; Trisomy 21; Partial Trisomy 21; Meiotic Nondisjunction; congenital heart defect; congenital cardiac malformation; cardiovascular malformation; congenital cardiac disease; congenital cardiac anomalies; congenital heart disease; Saudi Arabia; Kingdom of Saudi Arabia; KSA. No language constraints were used.

Study Selection Process

Two authors evaluated the titles, abstracts, or full texts of the search results to determine if they met the requirements for inclusion in this systematic review (we include all children with Down syndrome with no exclusions). Conflicts between the two authors were settled by discussion or agreement with a third author. If further information on possible research was required, email was utilized to contact the publication's linked author. All information relevant to the study topic was extracted from the included articles and entered into a Microsoft Excel Sheet (Microsoft Corporation, Redmond, WA).

Data Management

The primary search results were managed and duplicates were deleted via the Rayyan - Intelligent Systematic Reviews website (https://www.rayyan.ai/) [16]. After conducting title, abstract, and full-text screening, data were extracted from the included studies and entered into a Microsoft Excel spreadsheet.

Quality Assessment

Two authors used the Newcastle-Ottawa Scale (NOS) to rate the quality of the listed studies [17]. The study quality rating items have values ranging from 0 to 9. If a piece of research received seven or more stars, it was deemed to be of high quality.

Quantitative Data Synthesis and Analysis Plan

We used the MedCalc® Statistical Software version 20.115 (MedCalc Software Ltd., 2022, Ostend Belgium) for quantitative data synthesis [18]. A proportion random-effects meta-analysis included information on CHD prevalence. Forest and funnel plots were created. Higgin's I2 test was used to calculate the percentage of inter-study heterogeneity with an I2>50% cut-off selected as the threshold for significant heterogeneity. Egger’s test was used for assessing publication bias and a p-value less than 0.05 was the cut-off of publication bias. Begg’s test was used to assess if there was a significant correlation between the ranks of the effect estimates and the ranks of their variances. Funnel plots were utilized to visually assess publication bias.

Results

Search Results

**Figure 1:**
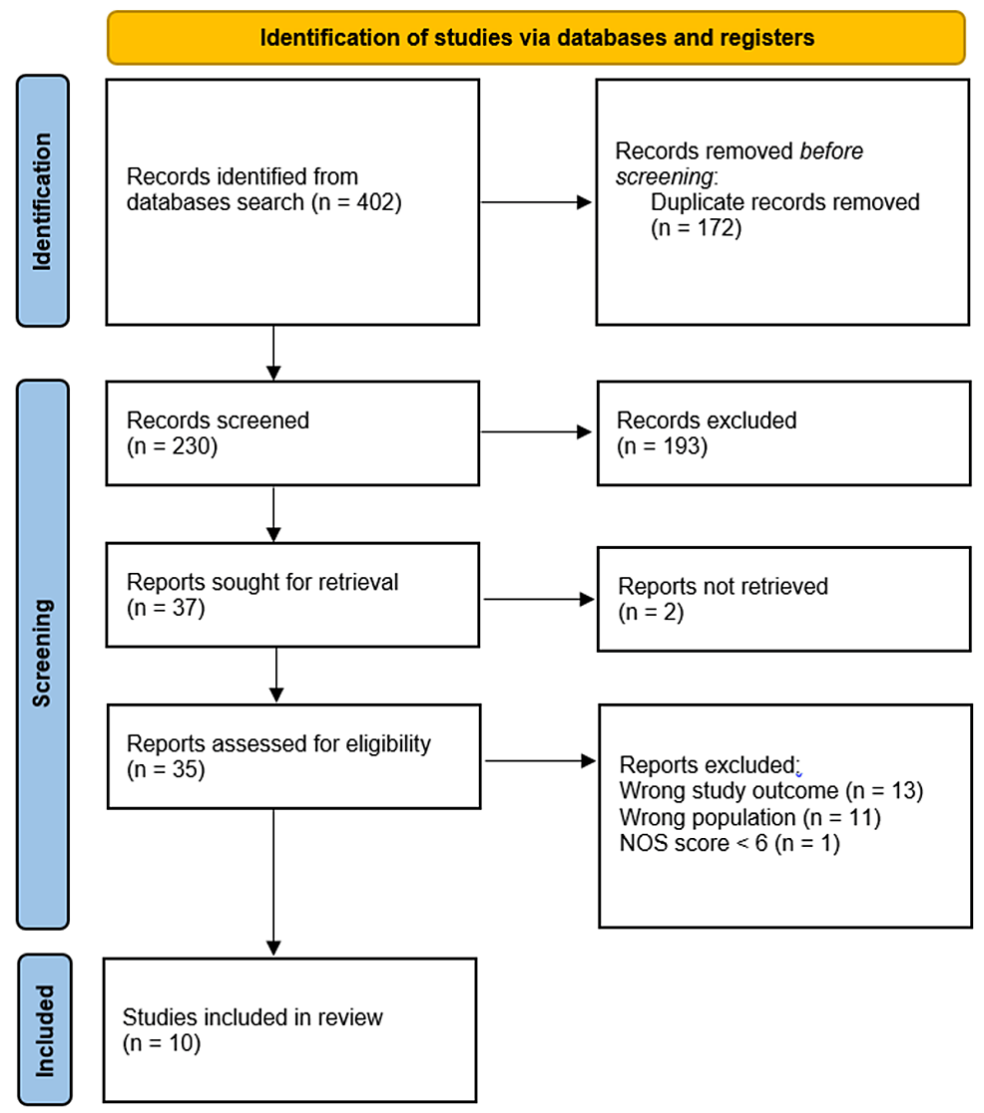
PRISMA flow chart for the summary of the search and screening processes PRISMA: Preferred Reporting Items for Systematic Reviews and Meta-Analyses

We initially retrieved a total of 402 citations from the primary search on the aforementioned electronic databases. The elimination of 172 studies due to duplicate detection and removal left 230 studies eligible for enrolment in the title and abstract screening. A total of 193 papers were eliminated for having unrelated objectives after the title and abstract screening. Ten articles were finally included after a full-text assessment of 35 studies. The search and study selection procedure is summarized in Figure 1.

Characteristics of the Included Studies

This review finally included 10 studies (Almawazini et al., 2017 [19], Abbag, 2006 [20], El-Attar, 2015 [21], Abduljawad et al., 2020 [22], Al-Aama et al., 2012 [23], Alhuzaimi et al., 2021 [24] Al-Jarallah, 2009 [25], Majeed-Saidan et al., 2019 [26], Morsy et al., 2016 [27], and Taura et al., 2021 [28]). A total of 1590 subjects were enrolled in the pooled analyses (Table 1). Male proportions ranged from 44% [19] to 57.1% [20]. The included population age ranged from 1 day to 33 years.

**Table 1:** Characteristics of the included studies NOS: not otherwise specified

Study	City	Design	Population	Study duration	Age, range	Males	Total	NOS
Abbag, 2006 [20]	Aseer	Retrospective	DS	1994-2005	-	57.10%	98	7
Abduljawad et al., 2020 [22]	Jeddah	Retrospective	DS	2005-2016	< 14 years	45.00%	129	7
Al-Aama et al., 2012 [23]	Jeddah	Prospective	DS	2007-2011	0-33 years	59%	130	8
Alhuzaimi et al., 2021 [24]	Riyadh	Retrospective	DS	2001-2019	0-18 years	54.90%	468	8
Al-Jarallah, 2009 [25]	Riyadh	Prospective	DS	2001-2004	1 day-12 years	56.40%	110	7
Almawazini et al., 2017 [19]	Albaha	Retrospective	DS	2010-2016	0-12 years	44.00%	150	7
El-Attar, 2015 [21]	Madinah	-	DS	2013-2015	2-12 years	53.40%	110	7
Majeed-Saidan et al., 2019 [26]	Riyadh	Prospective	DS	2010-2013	-		51	8
Morsy et al., 2016 [27]	Madinah	Retrospective	DS	2008-2013	-	50.30%	302	8
Taura et al., 2021 [28]	Bisha	Cross-sectional	DS	2016-2019	1 month-15 years	45.20%	42	7

Overall Prevalence of CHDs

**Figure 2:**
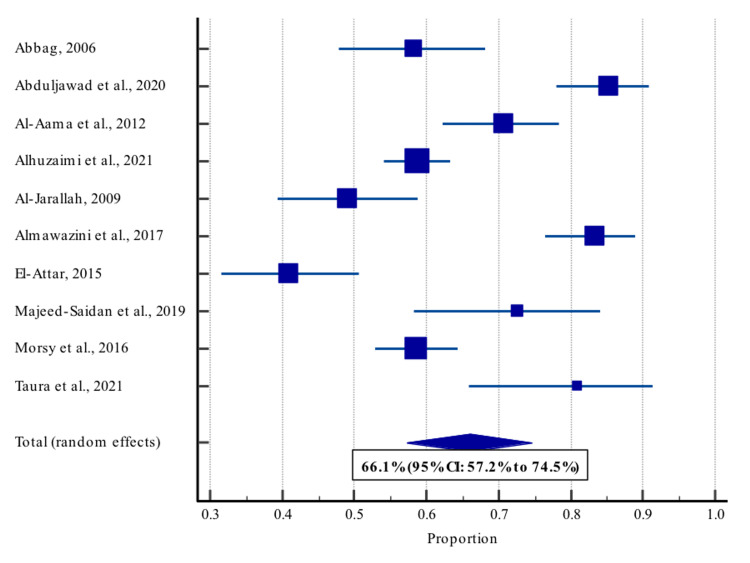
Forest plot showing the pooled prevalence of CHDs among individuals with DS CHD: congenital heart disease; DS: Down syndrome

**Figure 3:**
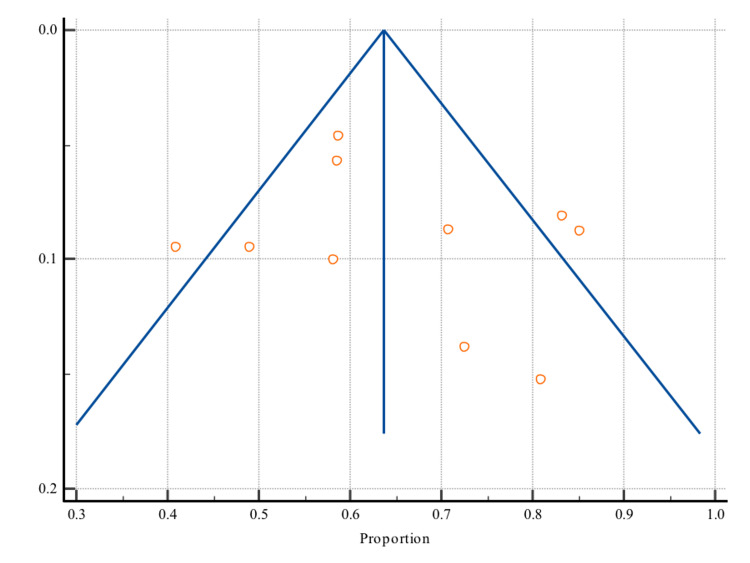
Funnel plot showing the symmetrical distribution of plotted prevalence data

A random-effects meta-analysis was conducted (Table 2; Figure 2) to estimate the pooled prevalence of CHDs in subjects with DS in KSA, and it showed a prevalence of 66.1% (95% CI: 57.2% to 74.5%). Table 3 shows that there is significant heterogeneity (I2 = 92.2%) and inspection of the funnel plot (Figure 3) shows the symmetrical distribution of plotted data. The prevalence rates ranged from 40.9% [21] to 85.3% [22].

**Table 2:** Quantitative prevalence data from the included studies

Study	Sample size	Proportion (%)	95% CI	Weight
Abbag, 2006 [20]	98	58.163	47.766 to 68.054	9.92
Abduljawad et al., 2020 [22]	129	85.271	77.959 to 90.893	10.20
Al-Aama et al., 2012 [23]	130	70.769	62.153 to 78.413	10.20
Alhuzaimi et al., 2021 [24]	468	58.761	54.150 to 63.260	10.89
Al-Jarallah, 2009 [25]	110	49.091	39.433 to 58.799	10.04
Almawazini et al., 2017 [19]	150	83.333	76.388 to 88.914	10.32
El-Attar, 2015 [21]	110	40.909	31.628 to 50.692	10.04
Majeed-Saidan et al., 2019 [26]	51	72.549	58.255 to 84.107	9.00
Morsy et al., 2016 [27]	302	58.609	52.827 to 64.221	10.74
Taura et al., 2021 [28]	42	80.952	65.882 to 91.399	8.64
Total (random effects)	1590	66.121	57.172 to 74.524	100.00

**Table 3:** Test for heterogeneity and publication bias

Q	115.3748
DF	9
Significance level	P < 0.0001
I^2^ (inconsistency)	92.20%
95% CI for I^2^	87.74 to 95.03
Egger's test	
Intercept	2.8734
95% CI	-4.9456 to 10.6924
Significance level	P = 0.4214
Begg's test	
Kendall's Tau	0.1348
Significance level	P = 0.5873

Discussion

According to international reports, CHD affects 40% to 63% of people with Down syndrome and is a key factor in morbidity and early death in these individuals [2,11,29,30]. According to various geographical regions throughout the globe, it has been reported that when describing the profiles and kinds of CHDs in DS, the major lesions detected vary [13,30]. Therefore, understanding the profile and features of CHD in DS for a certain nation is crucial in two ways: first, to increase survival by treating cardiac defects as soon as possible; and second, to implement the proper prevention measures. Respiratory infections have been implicated as the cause of mortality in both children and adults with DS, despite CHD being the recognized cause of the majority of early childhood deaths among patients with DS.

This systematic review and meta-analysis included data on 1539 subjects from 10 studies. The pooled prevalence of CHDs was found to be 66.1% (95% CI: 57.2% to 74.5%). However, there is significant heterogeneity (I2 = 92.2%) and inspection of the funnel plot shows the symmetrical distribution of plotted data.

This percentage is higher than that reported from Sudan (41%), Malaysia (49.3%), Sweden (54%), Japan (50.5%), Libya (45%), and Oman (60%), and lower than that reported in Morocco (100%), and Nigeria (78%) [31-35].

According to reports, ethnicity may have an impact on how often CHD occurs in children with Down syndrome [36]. An atrioventricular septal (AVSD) defect, for instance, is the CHD most often related to DS in western literature, followed by a ventricular septal defect (VSD), atrial septal defect (ASD), patent ductus arteriosus (PDA), and tetralogy of Fallot (TOF) [2]. On the other hand, a study from Korea found that ASD was the most prevalent defect, followed in decreasing order by VSD, PDA, and AVSD [37]. However, research from Pakistan found that VSD was the most typical abnormality, followed by PDA, AVSD, and TOF. Interestingly, PDA was the most prevalent single abnormality documented in Guatemalan research. Additionally, this was the concurrent cardiac defect that occurred most often together with other congenital cardiac diseases [38].

The following are the most prevalent CHDs in DS that have been reported in the international literature from western European nations and the USA: endocardial cushion defect (43%), which causes AVSD/AV canal defect; VSD (32%); secundum atrial septal defect (10%); tetralogy of Fallot (6%); and isolated PDA (4%). Multiple cardiac abnormalities are present in around 30% of individuals [2,11,29,39]. However, solitary VSDs have been shown to be the most prevalent defect in Asia, occurring in around 40% of patients [20]. In contrast, the majority of data from Latin America imply that the secundum form of ASD is the most frequent lesion [30,38].

## Conclusions

Our review found that 66% of subjects with DS in Saudi Arabia have at least one congenital heart defect. However, the reliability of our findings is limited due to the significant inter-study heterogeneity. We recommend further studies be conducted in order to provide higher-quality evidence to assess the prevalence of CHD.
